# Evaluation of Meibography Findings and Ocular Surface Parameters in Children with Atopic Dermatitis Without Eye Complaints

**DOI:** 10.3390/children12020150

**Published:** 2025-01-27

**Authors:** Şenol Sabancı, Hediye Küçükkeleş, Fatih Çelmeli, Sibel Yavuz

**Affiliations:** 1 Department of Ophthalmology, University of Health Science, Antalya Education and Research Hospital, 07100 Antalya, Turkey; drsibelakyol@hotmail.com; 2 Department of Pediatric Allergy and Immunology, University of Health Science, Antalya Education and Research Hospital, 07100 Antalya, Turkey; drhediyekucuk@hotmail.com (H.K.); fcelmeli@hotmail.com (F.Ç.)

**Keywords:** atopic dermatitis, meibography, ocular surface, dry eye, children

## Abstract

Background/Objectives: To evaluate the meibomian gland (MG) morphology and ocular surface parameters of children with atopic dermatitis (AD) without ocular symptoms. Materials and Method: Forty-five eyes of 24 children with AD and 44 eyes of 27 healthy children were included in the study. Upper and lower eyelid meibography measurements were performed for all cases and the rate of MG loss and the amount of tortuosity were evaluated. A Schirmer 1 test, tear break-up time (TBUT) test, and corneal staining scoring (CSS) were applied to all cases. Results: MG loss in the upper eyelid was 15.51 ± 10.39% in the AD group, while it was 9.30 ± 5.30% in the control group (*p* = 0.002). MG loss in the lower eyelid was 15.79 ± 10.93% in the AD group, while it was 9.23 ± 6.90% in the control group (*p* = 0.002). The rate of tortuosity in 26–50% of the upper and lower eyelid MGs was significantly higher in the AD group than in the healthy control group (*p* = 0.002 and *p* = 0.007, respectively). The Schirmer 1 test values were 10.98 ± 3.89 in the AD group and 17.41 ± 3.73 in the healthy control group (*p* < 0.0001). The TBUT values were found to be 9.18 ± 1.99 in the AD group and 11.40 ± 1.82 in the healthy control group (*p* < 0.0001). The CSS result was found to be significantly higher in the AD group than in the control group (*p* = 0.001). Conclusions: Significant MG loss was detected in patients with AD without eye complaints, which may cause the early deterioration of ocular surface parameters. We believe that close follow-up examinations of children with AD in terms of ocular surface complications may be necessary.

## 1. Introduction

Atopic dermatitis (AD) is a chronic, recurring skin disease characterized by itching, redness, and irritation of the skin [1]. Although the pathophysiology remains unclear, impaired skin barrier function, environmental and genetic factors, immune response imbalance, and alteration of the skin flora balance have been identified as the main causes [1,2]. AD is one of the most common skin diseases in children and usually occurs within the first five years of life. However, it can also occur at older ages [3]. Recent studies have shown that AD is associated with ophthalmic, gastrointestinal, nephrological, and other autoimmune diseases [4].

Meibomian gland (MG) dysfunction, blepharoconjunctivitis, dry eyes, cataracts, atopic keratoconjunctivitis, and keratoconus are ophthalmic disorders that are seen with increased frequency in patients with AD [5]. MGs are sebaceous glands arranged vertically in the lower and upper eyelids and are responsible for the synthesis of the lipid layer of tears [6]. MG dysfunction has been defined as a chronic disorder characterized by terminal duct obstruction and qualitative and quantitative changes in MG secretion [7]. Since MG dysfunction is seen with increased frequency in cases of AD, evaporative dry eye disease and posterior blepharitis due to decreased synthesis of the tear lipid layer are also observed more frequently [8].

This study aimed to compare the meibography findings and dry-eye parameters of children with AD without ophthalmic symptoms to those of healthy children without ophthalmic symptoms.

## 2. Method

The ethics committee approval for our study was obtained from the ethics committee of Antalya Training and Research Hospital (with decision number 15/16), and signed consent forms were obtained from the legal guardians of all participants.

Forty-five eyes of 24 children with AD who presented to our hospital’s pediatric allergy and immunology polyclinic and 44 eyes of 27 healthy children who presented to the ophthalmology department for routine examination were included in the study.

While creating both groups, the following exclusion criteria were applied: ophthalmic symptoms, a history of ocular surgery, vernal conjunctivitis, a history of contact lens use, dry eyes, chronic eye-drop use, prolonged use of periocular creams or ointments, systemic medication use that could cause MG dysfunction (e.g., antihistamines, antidepressants, and antiandrogens), being too young to comply with the tests, and poor-quality images. Patients with systemic diseases other than AD were excluded from the AD group, while those with any systemic diseases were excluded from the control group. Participants between the ages of 7 and 17 were included in the study.

All cases underwent a complete ophthalmologic examination, including visual acuity, biomicroscopic examination, intraocular pressure measurements (iCareIC100, rebound tonometry, Cal Coast Ophthalmic Instruments, Helsinki, Finland), and dilated fundus examination.

For tear break-up time (TBUT) measurement, OptiGlo (Wizcure Pharmaa-India) 1 mg sodium fluorescein strip was moistened with preservative-free artificial tears and placed in the lower fornix under a biomicroscope for 5 s and then removed. The patient was then asked to blink to ensure that the sodium fluorescein spread across the entire cornea. Under the cobalt blue light of the biomicroscope, the patient blinked twice and was then instructed not to blink again. The time until the first black spot appeared on the cornea was recorded. This procedure was repeated five times, and the TBUT was calculated as the average of the five measurements.

All cases underwent the Schirmer 1 test using topical anesthesia. Before the test, 0.5% proparacaine hidroklorür drops were instilled into the eyes. After waiting for 5 min, a Schirmer filter paper strip was folded at the tip and placed in the outer one-third of the lower eyelid fornix. The patient was instructed to close their eyes for 5 min. At the end of this period, the Schirmer filter paper was removed and the moist area was measured in millimeters from the folded part to determine the result.

**Figure 1:**
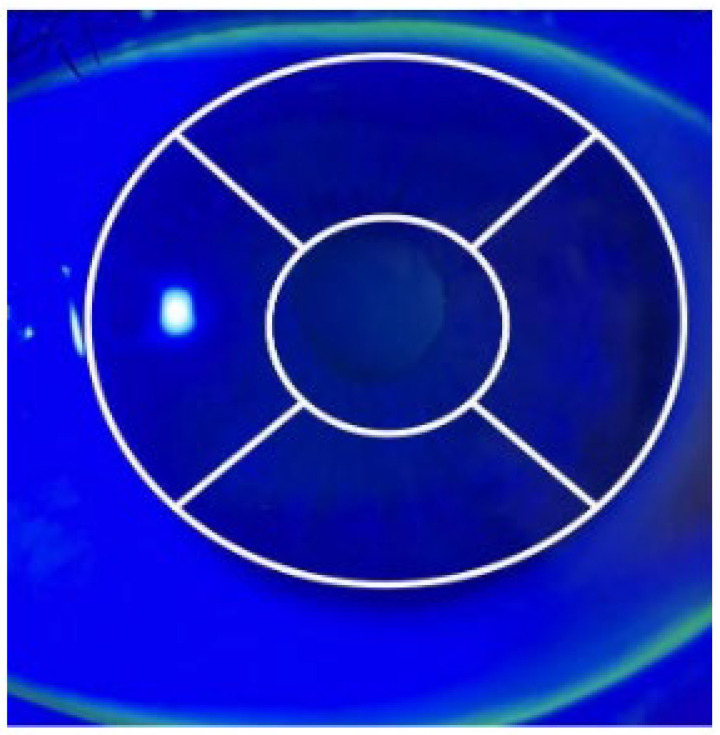
Corneal image showing the division of the cornea into five zones (central, superior, nasal, inferior, and temporal) for scoring corneal staining, as defined by the National Eye Institute.

The corneal fluorescence staining score was calculated for each case by dividing the cornea into five areas (central, inferior, superior, nasal, and temporal) based on the National Eye Institute reference (Figure 1). Each area was graded as follows: no staining (0 points), mild staining (1–15 punctate stains = 1 point), moderate staining (16–30 punctate stains = 2 points), and severe staining (≥30 punctate stains = 3 points). The points for all areas were summed to obtain the corneal staining score (CSS) for each eye [9].

The non-contact meibography images of both eyes were obtained using the Phoenix V.2.6 meibography imaging software, which uses infrared light and is integrated into the Sirius corneal topography (CSO, Florence, İtaly) device. Each patient was asked to place their chin on the designated part of the device and their forehead against the forehead support. The upper and lower eyelids were then everted sequentially, and five images were captured from each lid. The highest-quality images were selected for evaluation.

**Figure 2:**
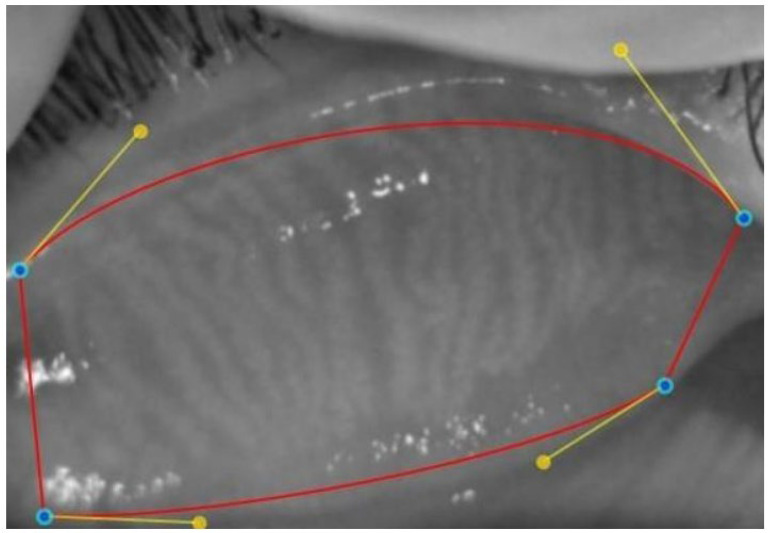
Manually defining the corners of the tarsus with blue dots and determination of the borders of the tarsus using the Phoenix meibography imaging software with red lines.

**Figure 3:**
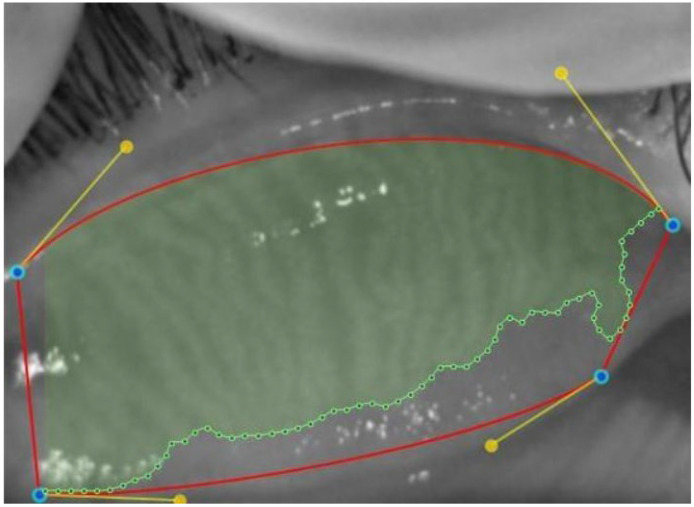
Manual determination of the meibomian gland area (Green area).

**Figure 4:**
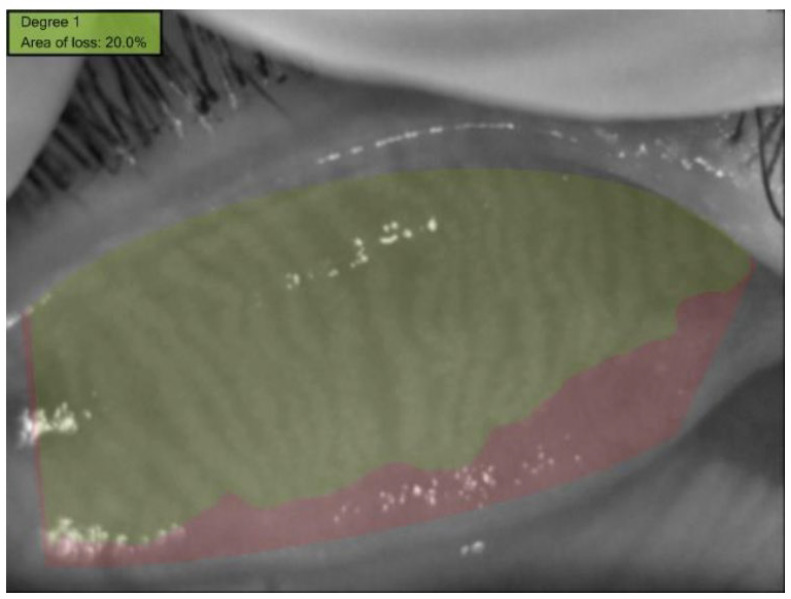
Automatic calculation of the meibomian gland loss rate using the Phoenix meibography imaging software. Green color shows MG area, red color shows Mg loss area.

In the selected upper and lower eyelid meibography images, the lid borders and the MG areas within these borders were marked using the Phoenix meibography software. The MG loss rate was automatically calculated and recorded (Figure 2, Figure 3 and Figure 4). The degree of MG loss was automatically categorized using the Meiboscale on the device as 0–9.99%, 10–25%, 26–50%, 51–75%, and ≥76% and was graded as 0, 1, 2, 3, and 4, respectively.

The MG tortuosity rate in the upper and lower eyelids was evaluated using the scale developed by Halleran according to this scale; tortuosity was defined as the presence of at least one MG area flexed 45° or more from the midline or multiple MG flexures of less than 45°. Tortuosity was graded as follows: grade 0; grade 1, 1–25% tortuosity; grade 2, 26–50% tortuosity; grade 3, 51–75%; and grade 4, 76–100% tortuosity [10,11,12].

### Statistical Analysis

Descriptive statistics were presented as the frequency, percentage, mean, standard deviation, median, minimum, maximum, and 25th–75th percentile values. For the categorical data analysis, a Fisher’s exact test was used if the percentage of cells with an expected value of less than 5 exceeded 20%, and the Pearson chi-square test was applied otherwise. Statistically significant differences between column ratios were evaluated using the z-test with a Bonferroni correction for multiple comparisons. The normality assumption was tested using the Shapiro–Wilk test. For numerical data, the Mann–Whitney U test was used to analyze differences between the two groups when data did not conform to a normal distribution. A non-parametric Spearman’s correlation coefficient was used to analyze the relationships between numerical variables. Statistical analyses were performed using SPSS version 23.0 software, with *p* < 0.05 being considered to be statistically significant.

## 3. Results

The mean age of the 24 children with AD who participated in the study was 9.91 ± 2.80 years, and the mean age of the 27 children in the healthy control group was 10.97 ± 3.18 years. No statistically significant difference was found between the two groups in terms of mean age (*p* = 0.11). Of the patients in the AD group, 15 were male (62.5%) and nine were female (37.5%), while 17 of the healthy controls were female (62.9%) and 10 were male (37.1%). There was no statistically significant difference between the female and male ratios in patients with and without AD (*p* = 0.069 < 0.05). A significant difference was found between the two groups in the grading of MG loss in the upper eyelid according to the Meiboscale. A statistically significant difference was observed between the two groups in the MG tortuosity rates in the upper and lower eyelids, as evaluated using the Hallerman scale (Table 1 and Table 2).

In the AD group, the MG loss rates in the upper and lower eyelids were significantly higher than in the control group. Schirmer 1 and TBUT test results were significantly lower in the AD group compared to the healthy control group (Table 3).

A statistically significant difference was found between the two groups in terms of corneal staining scores, which were higher in the AD group (Table 4).

A negative statistically significant correlation was observed between the MG loss rates in the upper and lower eyelids and the Schirmer 1 and TBUT test results. A positive statistically significant correlation was found between the MG loss rates in the upper and lower eyelids and the corneal staining score. Furthermore, a positive statistically significant correlation was detected between the Schirmer 1 and TBUT test results. Lastly, a statistically significant negative correlation was observed between the corneal staining score and both the Schirmer 1 and TBUT test results (Table 5).

## 4. Discussion

Studies investigating the underlying genetic causes of AD have an important place in the literature [13]. It has been reported that children with one parent who has AD are three times more likely to develop this condition and that children whose parents both have AD are five times more likely to develop it [14]. Many genes have been shown to be effective in the pathogenesis of AD. These genes are responsible for coding epidermal structural proteins. The most popular of these genes is the gene mutation that codes for the filaggrin protein, and it has been reported that mutations in this gene pose a very high risk for early-onset AD [15]. It has been shown that this filaggrin gene mutation may also occur in asymptomatic patients [16]. In addition to preventing fluid loss from the skin, the filaggrin protein also plays an important role in maintaining skin pH and preventing *Staphylococcus aureus* from overgrowing and advancing deeper into the skin [17]. In patients with AD, increased colonization of *S. aureus* has been detected as a result of the disruption of the skin barrier, supporting the above information, and it has also been found to be the most common agent causing infection [18]. MG dysfunction and anterior and posterior blepharitis are seen with increased frequency in AD cases [19,20]. Another study reported that the most common ocular morbidity was blepharitis in AD cases [21]. *S. aureus* is isolated in most anterior blepharitis cases and is characterized by crusting at the eyelash margin [19].

In clinical practice, MG dysfunction and posterior blepharitis are often intertwined concepts. In cases with MG dysfunction, posterior blepharitis is diagnosed clinically based on the observation of redness at the eyelid margin, telangiectatic vessels and meibomian cysts in the meibomian gland orifices [22]. MG dysfunction is triggered by the blockage of the gland orifices and the resulting interruption of meibomian secretion [23]. Unsecreted meibomian first causes dilatation in the central duct of the MG, then in the connector ductus and acini, followed by tortuosity and cystic degeneration in the entire gland, and finally gland loss.

Hyperkeratinization has been shown to be one of the causes of obstruction in the MG orifices, and hyperkeratinization has been reported frequently in cases of AD [24,25]. Another possible cause of obstruction in the MG orifices has been suggested to be *S. aureus* infection. It has been reported that the orifices may become blocked following the reaction that occurs after the triggering of inflammation and the migration of inflammatory cells to this area as a result of the toxins secreted due to increased *S. aureus* colonization at the eyelid margin; it has also been found that the cholesterol and fatty acids formed by the lipase enzyme secreted by the bacteria may cause the formation of obstruction in the orifices [26,27]. On the other hand, a cytological analysis of MG secretions has shown no inflammatory cells [24]. This may suggest that the inflammation is around the orifices rather than inside the gland, and this leads to fibrosis, causing long-term stenosis and obstruction in the orifices, and the mechanical pressure created by the meibomian accumulating in the gland and unable to exit from the orifices causes dilatation and tortuosity in the MGs, which is followed by MG loss. Squamous metaplasia is considered a precursor to squamous carcinoma and has been reported in some MGD cases. Squamous carcinoma has also been reported with increased frequency in AD cases in a study [28,29]. Squamous metaplasia may cause stenosis of the orifices in some AD cases, leading to MG loss.

In a prevalence study conducted by Gupta et al. in an asymptomatic and systemically healthy pediatric population, the rate of MG loss was reported to be 42% and the rate of tortuosity was 37% [30]. In our study, we found that the MG loss rates in the upper and lower eyelids were 36.3% and 40.9%, respectively, in the healthy control group. In our study, we found that MG loss rates were significantly higher in the lower and upper eyelids in the AD group. However, the reason why no significant difference was observed between the groups in the grading of lower eyelid MG loss according to the Meiboscale may be that the data were graded at certain intervals. In addition, studies have shown that lysozyme in tears is effective against Staphylococcus aureus [31]. When the distribution of tears on the ocular surface is examined, the lower tear meniscus height is found to be higher than the upper tear meniscus height [32]. This may cause less Staphylococcus aureus colonization at the lower eyelid margin, which may cause the MG loss to be relatively less visible in the meiboscalar of the lower eyelid. When examining tortuosity rates, we observed a significantly higher frequency of grade 2 tortuosity in the upper and lower eyelids of the AD group according to the Hallerman scale. We consider that this increased tortuosity rate may indicate future MG loss. In our study, we also observed that the MG loss rates in the upper and lower eyelids were significantly lower in the AD group than in the healthy control group.

The tear mucin layer is secreted by conjunctival goblet cells and ensures that the tear adheres to the corneal surface homogeneously for an extended period. The TBUT test, which is applied to establish or support the diagnosis of dry eye disease, evaluates tear film stability. Although this test is generally affected by the deficiency in the mucin component of the tear, some publications report that it is also affected in evaporative-type dry eye disease caused by a deficiency in the tear lipid component [33]. One study reported a decrease in conjunctival goblet cell density in patients with AD [34]. In MG disease, the disruption of tear integrity due to a deficient lipid component may cause a decrease in TBUT [35]. In our study, we also found that the TBUT test results were significantly lower in the AD group than in the control group.

One of the criteria for diagnosing dry eye disease is ocular surface staining [36]. In our study, the punctate corneal staining score was found to be significantly higher in patients with AD than in healthy controls. This may be due to the inability of the healthy tear film layer to adequately cover the cornea as a result of the impact on the MGs and conjunctival goblet cells. In addition, the decrease in tear secretion due to subclinical inflammation in patients with AD may also contribute to this situation. In fact, studies have reported that the Schirmer test measurements in patients with AD are lower than in healthy controls [37,38]. Although the Schirmer test mainly provides information about the amount of tears secreted from the lacrimal gland, it can also be affected by a decrease in the number of goblet cells, the inability of tears to adhere to the ocular surface due to a reduction in the tear mucin component, and increased evaporation caused by a deficiency in the tear lipid component. In the current study, we found that the Schirmer test measurements were significantly lower in patients with AD than in the healthy control group.

The limitations of our study include the small number of cases, the inability to establish a cause-and-effect relationship due to its cross-sectional design, the inability to measure the thickness of each of the mucus components and aqueous and lipid components of tears separately, the inability to perform a more objective non-invasive TBUT test and the inability to evaluate statistical power.

In conclusion, although the mean Schirmer values were within normal limits in asymptomatic children with AD, they were lower than those of the control group. In addition, the MG loss rates in the upper and lower eyelids were higher, and the TBUT values were lower, suggesting that the disease insidiously affects the eyes. Therefore, we consider that children diagnosed with AD should undergo more frequent ophthalmic examinations compared to their healthy peers due to the potential for ophthalmic complications.

## Figures and Tables

**Table 1:** Grading of upper eyelid MG loss according to Meiboscale and MG tortuosity rate according to the Hallerman scale.

		AD Group n (%)	Control Group n (%)	Total n (%)	*p*
MG loss in the upper eyelid according to the Meiboscale	Degree 0	13 ^a^ (28.9)	28 ^b^ (63.6)	41 (46.1)	0.004 ^1^
	Degree 1	26 ^a^ (57.8)	14 ^b^ (31.8)	40 (44.9)	
	Degree 2	6 ^a^ (13.3)	2 ^a^ (4.5)	8 (9)	
Hallerman tortuosity classification	0%	10 ^a^ (22.2)	5 ^a^ (11.4)	15 (16.9)	0.002 ^2^
	1–25%	21 ^a^ (46.7)	36 ^b^ (81.8)	57 (64)	
	26–50%	14 ^a^ (31.1)	3 ^b^ (6.8)	17 (19.1)	

^1^ Fisher’s exact test; ^2^ Pearson chi-square test. Different letters within the same row indicate statistically significant differences between column values (*p* < 0.05). MG: meibomian gland; AD: atopic dermatitis.

**Table 2:** Grading of lower eyelid MG loss according to the Meiboscale and MG tortuosity rate according to the Halerman scale.

		AD Group n (%)	Control Group n (%)	Total n (%)	*p*
MG loss in the lower eyelid according to the Meiboscale	Degree 0	19 (42.2)	26 (59.1)	45 (50.6)	0.122
	Degree 1	17 (37.8)	15 (34.1)	32 (36)	
	Degree 2	9 (20)	3 (6.8)	12 (13.5)	
Hallerman tortuosity classification	0%	20 ^a^ (44.5)	18 ^a^ (40.9)	38 (42.7)	0.007
	1–25%	15 ^a^ (33.3)	25 ^b^ (56,8)	40 (44.9)	
	26–50%	10 ^a^ (22.2)	1 ^b^ (2.3)	11 (12.4)	

Pearson chi-square test. Different letters within the same row indicate statistically significant differences between column values (*p* < 0.05). MG: meibomian gland; AD: atopic dermatitis.

**Table 3:** Comparison of MG loss rates, Schirmer 1 test values, and TBUT test values in the upper and lower eyelids between groups.

	AD Group (n: 45)		Control Group (n: 44)		*p*
	Mean ± SD (Min–Max)	Median (Q1–Q3)	Mean ± SD (Min–Max)	Median (Q1–Q3)	
Upper eyelid MG loss rate	15.1 ± 10.39 (1–40.8)	13.8 (8.7–21.2)	9.31 ± 5.84 (1.2–25.5)	7.2 (5.3–12.95)	0.002
Lower eyelid MG loss rate	15.79 ± 10.93 (1–42)	13.8 (6.6–21.6)	9.24 ± 6.91 (0–28.5)	7.1 (4.25–12.65)	0.002
Schirmer 1 test	10.98 ± 3.89 (1–19)	11 (9–13)	17.41 ± 3.73 (12–30)	17 (16–19)	<0.0001
TBUT test	9.18 ± 1.99 (6–14)	9 (7–11)	11.40 ± 1.82 (8–16)	12 (10–12)	<0.0001

Mann–Whitney U test. MG: meibomian gland; TBUT: tear break-up time; AD: atopic dermatitis.

**Table 4:** Comparison of corneal staining scores between groups.

		AD Group n (%)	Control Group n (%)	Total n (%)	*p*
Corneal staining score	0	6 ^a^ (13.3)	31 ^b^ (70.5)	37 (41.6)	<0.001
	1	11 ^a^ (24.4)	11 ^a^ (25)	22 (24.7)	
	2	24 ^a^ (53.3)	2 ^b^ (4.5)	26 (29.2)	
	3	4 ^a^ (8.9)	0 ^b^ (0)	4 (4.5)	
	Total	45 (100)	44 (100)	89 (100)	

Pearson chi-square test. Different letters within the same row indicate statistically significant differences between column values (*p* < 0.05). MG: meibomian gland, TBUT: tear break-up time, AD: atopic dermatitis.

**Table 5:** Correlations between Upper/Lower Eyelid MG Loss Rate, CSS, Schirmer 1 values, and TBUT.

	Schirmer 1	TBUT	Corneal Staining Score	Upper Eyelid MG Loss Rate	Lower Eyelid MG Loss Rate
TBUT test	0.680 **		−0.390 **	−0.158	−0.236 *
Corneal staining score	−0.582 **	−0.390 **		0.428 **	0.403 **
Upper eyelid MG loss rate	−0.289 **	−0.158	0.428 **		0.363 **
Lower eyelid MG loss rate	−0.388 **	−0.236 *	0.403 **	0.363 **	

Pearson’s correlation: * *p* < 0.05; ** *p* < 0.01; MG: meibomian gland, TBUT: tear break-up time.

## Data Availability

Data available on request due to restrictions (for ethical reasons).
